# Parental Lifetime PBSA Exposure Induces Neurodevelopmental Toxicity in F1 Zebrafish

**DOI:** 10.3390/toxics14070639

**Published:** 2026-07-22

**Authors:** Ruo Chen, Jie Chen, Junyan Tao, Wei Huang

**Affiliations:** 1 Pharmacy Department, Ruian Hospital of Traditional Chinese Medicine, Wenzhou 325200, China; ruoo_333@163.com (R.C.); 13676583946@163.com (J.C.); 2 School of Public Health, The Key Laboratory of Environmental Pollution Monitoring and Disease Control, Ministry of Education, Guizhou Medical University, No.6 Ankang Road, Guian New Area, Guiyang 561113, China

**Keywords:** PBSA, UV filter, zebrafish, intergenerational toxicity, neurodevelopmental toxicity

## Abstract

2-phenylbenzimidazole-5-sulfonic acid (PBSA) is a commonly used organic ultraviolet (UV) filter frequently found in aquatic environments, raising substantial ecological health concerns. While some toxic effects of PBSA on aquatic organisms have been reported, its intergenerational developmental and neurotoxic risks remain poorly understood. In this study, we established a zebrafish life-cycle exposure model to explore the intergenerational toxicity of environmentally relevant concentrations of PBSA (0.2, 2, and 20 μg/L). Offspring were categorized into three exposure groups: parental exposure only (F0+/F1−), parental exposure with continuous F1 exposure (F0+/F1+), and only F1 exposure without parental treatment (F0−/F1+). Our findings demonstrate the transfer of PBSA from parental gonads to F1 embryos. Parental lifetime exposure significantly inhibited somitogenesis and increased mortality and malformation rates in the F1 generation, with the most pronounced developmental damage observed in the F0+/F1+ group. Whole-mount immunohistochemistry revealed that PBSA notably reduced motor neuron axon length in F1 larvae, accompanied by downregulation of developmental-related genes including *gap43*, *mbp*, and *shha*. Mechanistically, the F0+/F1− group exhibited a marked increase in MDA levels. Excessive ROS accumulation and reduced CAT activity were specifically observed in the F0+/F1+ group, whereas the F0+/F1− and F0−/F1+ groups showed significantly elevated CAT activity, jointly driving developmental and neurotoxic changes. In silico predictions indicated low acute aquatic toxicity of PBSA, contradicting its observed intergenerational risks. Our findings demonstrate that parental lifetime PBSA exposure can induce significant intergenerational neurodevelopmental toxicity in zebrafish offspring by disrupting oxidative balance and neural gene expression, with continuous offspring exposure further aggravating the adverse effects. This research underscores that traditional single-generation toxicity assessments underestimate the ecological dangers of UV filters. It also offers new insights into the environmental risk evaluation of PBSA and similar emerging contaminants.

## 1. Introduction

2-phenylbenzimidazole-5-sulfonic acid (PBSA) is a widely used hydrophilic organic UV filter in sunscreen and personal care products due to its excellent UV absorption capacity and high water solubility [1,2]. However, PBSA shows poor removal efficiency in wastewater treatment systems, with an average removal rate of only 11 ± 36%, making it one of the most frequently detected UV filters in aquatic environments worldwide [1,2,3,4]. It has been detected at concentrations of up to 4560 ng/L in wastewater effluents across Australia, and up 170 ng/L in estuarine and coastal waters of the Baltic Sea [1,2], indicating persistent environmental exposure contamination. Furthermore, field monitoring in South Bohemia revealed tiered PBSA levels across various aquatic environments, with concentrations ranging from 5.1 to 48 ng/L in pristine surface waters, 11 to 500 ng/L in rivers affected by wastewater, and 24 to 930 ng/L in recreational ponds. The highest concentration observed was 13,000 ng/L (13 μg/L) in heavily frequented outdoor swimming pools [5]. Given that PBSA can enter organisms through dermal contact, drinking water, and dietary intake, its potential long-term toxic effects on aquatic organisms and human health have raised widespread concerns.

Despite the widespread presence of PBSA in aquatic environments, its desulfonated and brominated transformation byproducts exhibit higher toxic potential [6], while its biological and neurodevelopmental risks remain inadequately characterized. Recent studies have revealed that juvenile zebrafish exposed to 0.5 and 5 mg/L of PBSA for 7 days suffered dose-dependent hepatic oxidative damage, as evidenced by suppressed antioxidant enzyme (SOD) activities, a perturbed detoxification system (GST and GSH), and a marked increase in the lipid peroxidation end-product MDA [7]. Our previous study demonstrated that lifetime exposure to environmentally relevant concentrations of PBSA significantly disrupts the hypothalamic–pituitary–gonadal–liver (HPGL) axis, inhibits ovarian maturation, reduces fecundity, and impairs embryonic development in zebrafish [8]. However, these studies mainly focus on direct exposure effects, while the potential intergenerational developmental and neurotoxicity of PBSA remain unclear.

In aquatic environments, offspring may be affected by parental exposure to environmental pollutants, and such intergenerational effects can exacerbate developmental toxicity in the absence of direct chemical exposure in early life stages [9,10,11]. Fish embryos are highly vulnerable to environmental chemicals during the early stage, such as 24 hpf, a critical period for zebrafish neural and somite development [12,13]. Exposure at this stage can trigger molecular disruptions, including endocrine disruption and redox imbalance, leading to persistent developmental abnormalities and increased larval mortality. Although the intergenerational developmental toxicity of various environmental contaminants has been gradually recognized, the specific mechanisms by which parental PBSA exposure interferes with oxidative stress and neural development in F1 offspring remain largely unknown. Notably, no published studies have yet characterized PBSA-induced neurological damage across generations. Therefore, it is essential to conduct a thorough assessment of the neurodevelopmental effects of PBSA on progeny after parental exposure in order to comprehend the biological consequences of this UV filter.

Building upon our previous findings indicating that lifetime exposure to PBSA induces disruptions in reproductive endocrine function and impairs offspring development in zebrafish, the current investigation delves into the intergenerational effects of PBSA on development and neurotoxicity. Zebrafish (F0) were exposed to concentrations of 0.2, 2, and 20 μg/L of PBSA continuously from the embryonic stage (6 hpf) to sexual maturity (5 months). Subsequently, fertilized F1 embryos were collected and either continued to be exposed to PBSA or raised in toxicant-free water, allowing us to distinguish parental transfer effects from direct embryonic exposure. We systematically evaluated various toxicological developmental endpoints in F1 embryos, analyzed the transcript levels related to neural development to characterize neurotoxicity, and explored the involvement of oxidative stress. This study enhances our comprehension of the environmental risk assessment of UV filters on F1 embryos following parental exposure.

## 2. Materials and Methods

### 2.1. Zebrafish Maintenance and Embryo Harvesting

Wild-type AB strain zebrafish were used in this study. Adult fish were housed in a system under standardized conditions: water temperature maintained at 28 ± 1 °C, pH 7.0, conductivity ranging from 500 to 1000 μS/cm, and a 14 h light/10 h dark photoperiod. The Fish were fed twice daily with live brine shrimp. On the day prior to spawning, healthy adults were transferred into breeding tanks at a female-to-male ratio of 1:1. Spawning was induced by light stimulation the following morning, and eggs were harvested within 30 min. The collected embryos were rinsed thoroughly with embryo medium (EM). Healthy embryos with normal developmental morphology were selected under a microscope at 6 h post-fertilization (hpf) for subsequent exposure experiments, following established zebrafish staging protocols [14]. The research was approved by the Institutional Animal Care and Use Committee of Guizhou Medical University, with the experimental protocol registered under permit number 2100215.

### 2.2. In Silico Toxicity Prediction Using ECOSAR

The acute aquatic toxicity and toxicological properties of PBSA were predicted using the ECOlogical Structure–Activity Relationship (ECOSAR) model version 2.2 (https://episuite.dev/EpiWebSuite/#/, accessed on 10 July 2025) and Toxtree software version 3.2.0 (http://toxtree.sf.net/predict, accessed on 15 July 2025). PBSA was assigned to the neutral organics baseline structural class for calculation because its molecule contains benzenesulfonic acid groups and no sulfonamide moieties. Based on the molecular structure of PBSA, ECOSAR automatically calculated predicted 96 h LC_50_ for fish and 48 h LC_50_ for Daphnia magna, as well as 96 h EC_50_ for green algae. The predicted no-effect concentration was derived by dividing the lowest predicted acute toxicity value by an assessment factor of 1000, and the risk quotient (RQ) was further calculated to evaluate the potential environmental risk of PBSA at environmentally relevant concentrations. Toxtree was used to assess toxicity-related alerts, including mode of action classification, Cramer rules, structural alerts for genotoxicity, carcinogenicity, skin sensitization, and protein/DNA binding potential, as well as the Kroes TTC decision tree for safety concern evaluation.

### 2.3. Chemical Stock Solutions and Exposure Procedure

PBSA (2-phenylbenzimidazole-5-sulfonic acid, CAS: 27503-81-7, 96% purity) was purchased from Sigma-Aldrich, St. Louis, MO, USA. Stock solutions were prepared in dimethyl sulfoxide (DMSO, Sigma-Aldrich), and stored at 4 °C in the dark following our previously published research [8]. In short, a 200 μg/mL stock solution of PBSA was prepared in dimethyl sulfoxide (DMSO). The final volume percentage of DMSO was precisely adjusted to 0.01% in both the solvent control and all PBSA-treated groups to prevent solvent-induced toxic bias. Working solutions were freshly diluted from the stock immediately before use. F0 zebrafish (5 months old) were randomly assigned to four treatment groups: solvent control (0.01% DMSO) and PBSA at nominal concentrations of 0.2, 2, and 20 μg/L. The chosen concentrations were based on field surveillance data, which showed PBSA levels of approximately 0.17 μg/L in coastal water, 4.56 μg/L in wastewater, and reaching up to 13 μg/L in heavily used outdoor swimming pools. This selection accounts for current environmental conditions and potential future episodes of increased pollution [1,2,5]. Each group comprised three replicate tanks, each containing 10 fish (5 males and 5 females). The exposure duration was 150 days (from 6 hpf to 5 months old), with daily replacement of exposure solutions. Following the F0 exposure period, all fish (both exposed and the control) were moved to PBSA-free water for a 12 h washout interval before spawning induction. Light stimulation induced spawning the next morning, and healthy F1 embryos were harvested within 30 min. Embryos from each mating pair were randomly split and equally distributed to all F1 experimental cohorts to avoid clutch-specific bias. After rinsing three times with EM, F1 embryos from the exposed parent were separate into two cohorts. One subset was continually exposed to the identical PBSA concentrations as their progenitors (F0+/F1+), while the other subset was raised in clean EM (F0+/F1−). Simultaneously, embryos from the unexposed parental group were either cultured in PBSA-free EM (F0−/F1−) or exposed to the corresponding PBSA concentrations (F0−/F1+).

### 2.4. Developmental Toxicity Assessment of PBSA in F1 Zebrafish Embryos

Developmental endpoints were assessed in F1 embryos according to our previously established protocols [11,15]. Specifically, cumulative somite count, cumulative mortality, and cumulative malformation rates were assessed at 13, 24, and 96 hpf. Each treatment group consisted of 50 F1 embryos *per* replicate (three replicates *per* concentration) placed in a 90 mm petri dish with 30 mL of the corresponding exposure solution. Daily renewal of exposure solutions was performed, and mortalities were recorded and removed promptly. Given the severe developmental defects observed at 20 μg/L, this concentration was chosen for subsequent neurodevelopmental and oxidative stress mechanistic analyses, consistent with our previously published study [8].

### 2.5. Liquid Chromatography–Mass Spectrometry (LC-MS)

Prior to the quantification of tissue PBSA, LC-MS was applied to verify PBSA stability in exposure medium over a 24 h renewal cycle, and negligible concentration loss was observed during the test. PBSA content in zebrafish was measured using a previously established LC-MS method [11,15]. Concentrations of PBSA in F0 gonadal tissues and F1 embryos were determined by LC-MS analysis. For F0 adults, ovarian tissues were paired as one sample and testicular tissues were pooled (five testes *per* sample), with three biological replicates established. For F1 embryos at 2 hpf, 200 individuals were collected *per* replicate, with a total of three replicates. Detailed procedures for sample extraction, pretreatment, instrumental conditions, and method validation can be found in the Supplementary Materials Text S1.

### 2.6. Whole-Mount Immunohistochemistry

To assess the potential neurodevelopmental effects of PBSA on motor neuron development in F1 larvae, we conducted whole-mount immunohistochemistry with the motor neuron-specific antibody Znp1 (DSHB, Iowa City, IA, USA, RRID: AB_2315626) following methods outlined in our prior work [16]. Briefly, F1 larvae at 48 hpf were dechorionated and fixed at 4 °C overnight in 4% paraformaldehyde (Solarbio, Beijing, China). Subsequently, larvae underwent a series of washes in PBST, dehydration in methanol (Hongsheng Fine Chemical Co., Ltd., Changshu City, China), and rehydration. Antigen retrieval involved a 20 min incubation in 150 mM Tris-HCl (Solarbio, Beijing, China, pH = 8.8). Following blocking in 10% goat serum (Gibco, New York, NY, USA) in PBT (PBS with 0.8% Triton X-100) for 1 h at room temperature, larvae were incubated overnight at 4 °C with the primary antibody Znp1 (1:250, DSHB). After three washes with PBST, larvae were incubated with Alexa Fluor 488-conjugated goat anti-mouse secondary antibody (1:500, Abcam, Cambridge, UK) for 1.5 h at room temperature in the dark. Stained larvae were mounted in 70% glycerol/PBS and imaged using a fluorescence microscope. For axon quantification, three intact primary motor axons anterior to the cloaca were randomly selected from each larva, and the mean length of the three axons was calculated as the representative axon length *per* individual. Axon length of primary motor neurons was quantified with Image J 1.51 software following our previously established protocol [16]. Each treatment group underwent analysis of 15 larvae *per* biological replicate (three replicates).

### 2.7. Quantitative Real-Time PCR (qPCR)

The qPCR assay was performed according to our previously established protocol [12]. Using TRIzol reagent (Invitrogen, Carlsbad, CA, USA), total RNA was isolated from whole F1 larvae at 48 hpf, which was strictly conducted in accordance with the manufacturer’s recommended procedures. Assessment of RNA concentration and purity was conducted via spectrophotometer analysis, while 1% agarose gel electrophoresis was employed to verify RNA integrity. Purified total RNA was reversely transcribed into cDNA using the FastQuant cDNA Synthesis Kit (Tiangen, Beijing, China), which includes an integrated DNase I digestion step to remove residual genomic DNA before reverse transcription. Subsequent quantitative real-time PCR analysis was performed on a PCR Detection System (Bio-Rad, Hercules, CA, USA). The reaction procedure was set as an initial pre-denaturation at 95 °C for 30 s, and then 40 amplification cycles of 95 °C for 5 s and 60 °C for 30 s. A melting curve program was run at the end of amplification to verify the specificity of the PCR products. Two negative controls including NTC (nuclease-free water instead of cDNA template) and NRT (RNA without reverse transcriptase) were included in each qPCR plate. Primer sequences for target genes (*gap43*, *mbp*, *shha*, and *α1-tubulin*) were retrieved from our previously published work [15], as listed in Table 1. Gene expression levels were quantified using the method after normalization to β-actin. Three independent biological replicates were prepared *per* treatment group, and each cDNA sample was run with three technical replicates in qPCR to ensure data reproducibility.

### 2.8. Redox Analysis

To evaluate the redox status of zebrafish under different PBSA exposure regimes, intracellular levels of ROS, MDA and CAT activity were determined following our previously described methods [16,17].

For the ROS assay, larvae at 48 hpf were first dechorionated using 100 μg/mL of protease E (Roche, Mannheim, Germany) at 28 °C before staining, and then incubated with 30 μM of DCFH-DA (Nanjing Jiancheng, Nanjing, China) dissolved in EM for 30 min in darkness (8 embryos *per* replicate, 3 replicates). After incubation, embryos were anesthetized with 0.02% MS-222 (Sigma-Aldrich, St. Louis, MO, USA), and full lateral whole body fluorescence images were captured using a fluorescence microscope. The average fluorescence intensity was analyzed using Image J software.

For biochemical analysis, zebrafish (60 embryos *per* replicate, 3 replicates) were homogenized in ice-cold PBS and centrifuged at 10,000× *g* for 10 min at 4 °C. The supernatants were collected for subsequent measurements. MDA content, indicative of lipid peroxidation, was assessed utilizing the thiobarbituric acid (TBA) method with a commercial kit (Nanjing Jiancheng, Nanjing, China). CAT activity was gauged by monitoring the decomposition of H_2_O_2_ at 405 nm per the manufacturer’s instructions (Nanjing Jiancheng, China). MDA levels were normalized to total protein content and expressed as nmol/mg protein, while CAT activity was denoted as U/mg protein.

### 2.9. Statistical Analysis

Statistical calculations were carried out using GraphPad Prism 8.0 software. Before statistical comparison, the Kolmogorov–Smirnov test was used to evaluate the normal distribution of raw data, and Levene’s test was applied to detect the homogeneity of variance among groups. For comparisons between two independent experimental groups, the unpaired Student’s *t*-test was adopted. Differences across multiple treatment groups were analyzed by one-way analysis of variance (ANOVA), followed by Tukey’s multiple comparison post hoc test for pairwise comparisons. The results are expressed as mean ± standard deviation (SD), with statistical significance defined as *p* < 0.05.

## 3. Results

### 3.1. In Silico Toxicity Prediction of PBSA

ECOSAR prediction showed that the LC_50_ of PBSA was 1.96 × 10^5^ mg/L for fish, 9.12 × 10^4^ mg/L for Daphnia magna, and 2.98 × 10^4^ mg/L for green algae. The calculated PNEC was higher than the tested concentration (20 μg/L), with RQ < 0.1, indicating insignificant acute aquatic toxicity. Toxtree analysis classified PBSA as Cramer Class I (low concern) and showed no mutagenicity alerts. Mild structural alerts were observed for potential micronucleus formation and non-genotoxic carcinogenicity, but no reactivity alerts for Michael addition or genotoxic carcinogenicity were found (Table 2).

### 3.2. Developmental Indicators of Parental PBSA Exposure on F1 Zebrafish

In order to confirm the transfer of PBSA from maternal and paternal sources, PBSA concentrations were measured in F0 ovaries, F0 testes, and 2 hpf F1 embryos across four exposure groups. PBSA was not detected in the control samples, while quantifiable residues of PBSA were present in gonads and F1 embryos at all PBSA treatment levels. At the highest exposure level of 20 μg/L, PBSA concentration in ovaries was 1.81 ± 0.03 μg/mg, exceeding the level in testes (1.24 ± 0.08 μg/mg) at the same exposure, indicating higher accumulation of PBSA in ovaries. Additionally, trace amounts of PBSA were found in 2 hpf F1 embryos from parental exposure of 0.2 μg/L, with PBSA levels in embryos increasing with higher parental exposure concentrations (Table S1).

**Figure 1:**
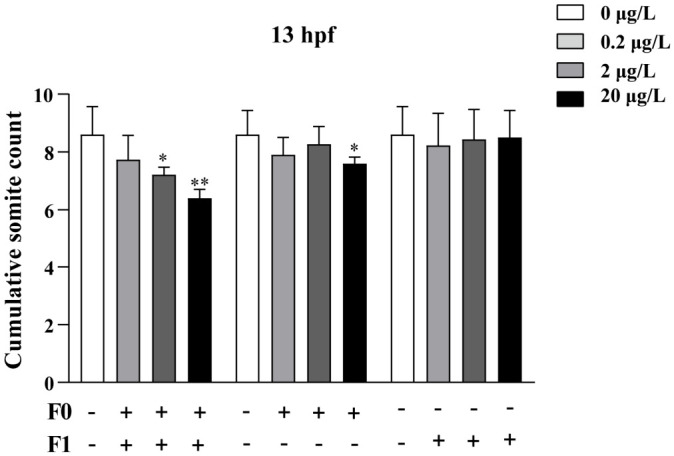
Effects of PBSA on cumulative somite count in F1 zebrafish embryos at 13 hpf. Three exposure scenarios are presented: PBSA-exposed parents with continuous F1 exposure (F0+/F1+), parental exposure alone (F0+/F1−), and only F1 exposure without parental treatment (F0−/F1+). Values are expressed as mean ± SD. Asterisks denote significant differences compared with the control group within the same exposure scenario (* *p* < 0.05, ** *p* < 0.01).

At 13 hpf, cumulative somite counts significantly reduced in F1 embryos (F0+/F1+) at 2 μg/L (*p* < 0.05) and 20 μg/L (*p* < 0.01), as well as in the F0+/F1− group at 20 μg/L (*p* < 0.05), compared to the control. No notable changes were evident in the F0−/F1+ group. Moreover, the inhibitory effect on somitogenesis was more severe in the F0+/F1+ group compared to the F0+/F1− group at 20 μg/L, indicating aggravated developmental inhibition under combined parental and F1 exposure (Figure 1).

**Figure 2:**
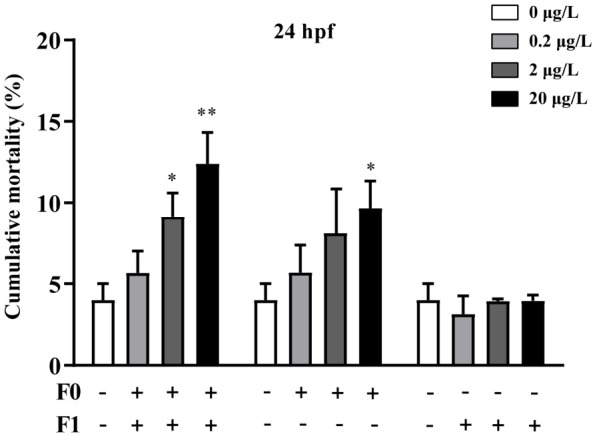
Effects of PBSA on cumulative mortality of F1 zebrafish embryos at 24 hpf. Three exposure scenarios are presented: PBSA-exposed parents with continuous F1 exposure (F0+/F1+), parental exposure alone (F0+/F1−), and only F1 exposure without parental treatment (F0−/F1+). Values are expressed as mean ± SD. Asterisks denote significant differences compared with the control group within the same exposure scenario (* *p* < 0.05, ** *p* < 0.01).

At 24 hpf, cumulative mortality significantly increased in the F0+/F1+ group (*p* < 0.05 for 2 μg/L, *p* < 0.01 for 20 μg/L), as well as in the F0+/F1− group at 20 μg/L (*p* < 0.05), compared to the control. Conversely, no significant mortality increase was evident in embryos from the F0−/F1+ group at any concentration. Moreover, the lethal effect of PBSA was more severe in the F0+/F1+ group than in the F0+/F1− group at 20 μg/L, showing intensified lethal damage when parents and offspring were both exposed to PBSA (Figure 2).

**Figure 3:**
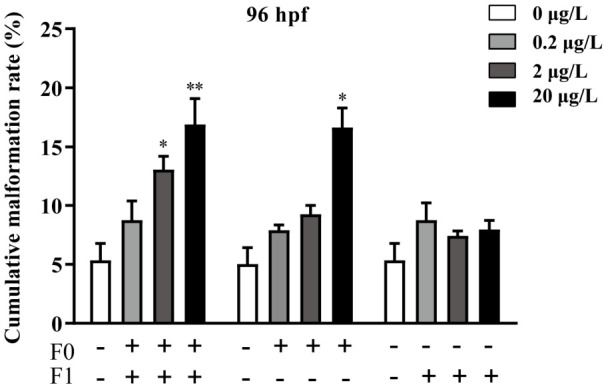
Effects of PBSA on the cumulative malformation rate of F1 zebrafish larvae at 96 hpf. Three exposure scenarios are presented: PBSA-exposed parents with continuous F1 exposure (F0+/F1+), parental exposure alone (F0+/F1−), and only F1 exposure without parental treatment (F0−/F1+). Values are expressed as mean ± SD. Asterisks denote significant differences compared with the control group within the same exposure scenario (* *p* < 0.05, ** *p* < 0.01).

At 96 hpf, cumulative malformation rates significantly increased in the F0+/F1+ group at 2 μg/L (*p* < 0.05) and 20 μg/L (*p* < 0.01), and in the F0+/F1− group at 20 μg/L (*p* < 0.05). Conversely, no significant increase in malformation rate was noted in the F0−/F1+ group (Figure 3).

### 3.3. Effects of PBSA on Axon Length and Neurodevelopment-Related Gene Expression in F1 Zebrafish

**Figure 4:**
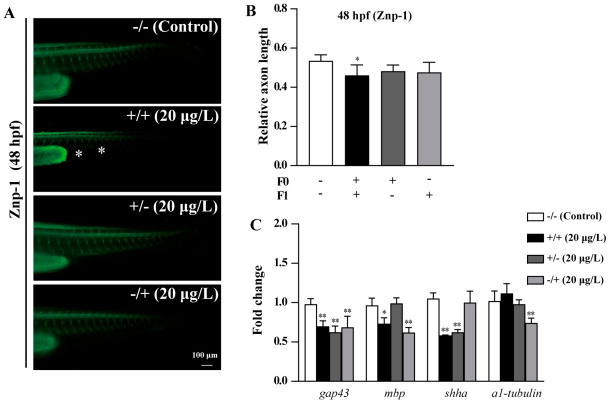
Effects of 20 μg/L of PBSA on axonal development and neurodevelopment-related gene expression in F1 zebrafish. (**A**) Representative images of motor neuron axonal labeled by Znp-1 immunofluorescence at 48 hpf. Scale bar: 100 μm. White asterisks signify discontinued axonal growth. (**B**) Quantification of relative axon length at 48 hpf. (**C**) Relative mRNA expression levels of neurodevelopment-related genes (*gap43*, *mbp*, *shha*, and *α1-tubulin*). Different exposure scenarios are presented: F0−/F1− (Control), F0+/F1+, F0+/F1−, and F0−/F1+. Values are expressed as mean ± SD. Asterisks denote significant differences compared with the control group (* *p* < 0.05, ** *p* < 0.01).

At 48 hpf, motor neuron axonal development and the expression of neurodevelopment-related genes were examined in F1 larvae following 20 μg/L PBSA exposure. Immunofluorescence staining with Zn-1 showed that relative axon length was markedly reduced in the F0+/F1+ group compared to the control (*p* < 0.05), with no notable changes in the F0+/F1− or F0−/F1+ groups (Figure 4A,B). For neurodevelopment-related gene expression, significant downregulation was detected in the F0+/F1+ group for *gap43*, *mbp*, and *shha* (*p* < 0.05 or *p* < 0.01). The F0+/F1− group revealed a significant downregulation of *gap43* and *shha*, whereas the F0−/F1+ group exhibited reduced expressions of *gap43*, *mbp*, and *α1-tubulin* (Figure 4C). These results indicate that F0+/F1+ exposure to 20 μg/L PBSA significantly impaired axonal development and altered the expression of key neurodevelopmental genes.

### 3.4. Effects of PBSA Exposure on Oxidative Stress in F1 Zebrafish Larvae

**Figure 5:**
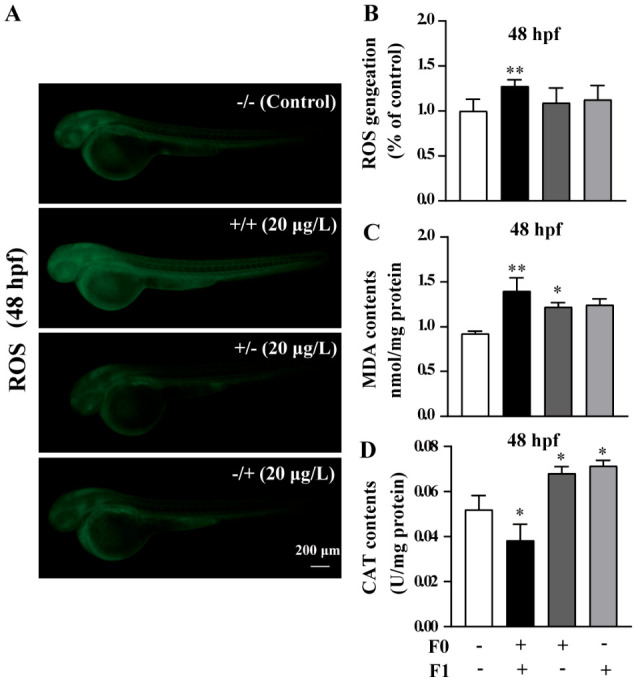
Effects of 20 μg/L of PBSA on oxidative stress indicators in F1 zebrafish larvae at 48 hpf. (**A**) Representative images of ROS generation detected by DCFH-DA staining (scale bar: 200 μm). (**B**) Quantification of ROS generation (% of control). (**C**) MDA content (nmol/mg protein). (**D**) CAT activity (U/mg protein). Three exposure scenarios are presented: F0−/F1− (control), F0+/F1+, F0+/F1−, and F0−/F1+. Values are expressed as mean ± SD. Asterisks denote significant differences compared with the control group (* *p* < 0.05, ** *p* < 0.01).

At 48 hpf, ROS generation notably increased in the F0+/F1+ group compared to the control (*p* < 0.01), with no significant changes in the F0+/F1− or F0−/F1+ groups (Figure 5A,B). Elevated MDA contents were observed in the F0+/F1+ (*p* < 0.01) and F0+/F1− (*p* < 0.05) groups (Figure 5C). CAT activity significantly decreased in the F0+/F1+ group (*p* < 0.05), while the F0+/F1− and F0−/F1+ groups exhibited significant increases (*p* < 0.05) (Figure 5D).

## 4. Discussion

Distinct from our previous research that only investigated reproductive endocrine disturbance with single parental PBSA exposure, this study established a model incorporating both lifetime F0 parental exposure and continuous F1 larval exposure. We systematically assessed developmental abnormalities and motor neuron axonal impairment in the offspring, identifying oxidative stress imbalance as a remarkably correlated toxic phenotype concurrent with intergenerational neurodevelopmental defects. In the present study, using a full life-cycle exposure model in zebrafish, we investigated the intergenerational neurodevelopmental toxicity of PBSA by comparing three distinct exposure scenarios in the F1 generation. Our results demonstrate that parental lifetime exposure to environmentally relevant concentrations of PBSA significantly impairs early development in F1 offspring, and these effects are further exacerbated by continuous PBSA exposure in F1 larvae. By contrast, direct F1 exposure without parental treatment caused only minor or insignificant effects. These findings confirm the potent intergenerational toxicity of PBSA and highlight the dominant role of parental exposure in mediating developmental disorders in offspring.

Parental exposure to environmental contaminants has been increasingly recognized for inducing intergenerational developmental toxicity in offspring, even without direct embryonic exposure [9,10,11]. Our previous research detected blocked ovarian maturation via ovarian hematoxylin–eosin (H&E) staining and also confirmed that PBSA disrupts the HPGL axis and reduces sex hormone levels in zebrafish [8]. Based on the published gonadal histopathological and endocrine data, we hypothesize that the intergenerational neurodevelopmental toxicity induced by PBSA may predominantly result from parental reproductive impairment and compromised gamete quality rather than direct waterborne exposure to offspring. This interpretation is a speculative extrapolation, given the absence of gonadal or gamete assays in this study. Similarly, Cao’s research on azoxystrobin indicated that parental endocrine disruption and gamete abnormalities are key factors in developmental anomalies in the F1 generation [18]. Analysis of PBSA concentrations in F0 gonads and F1 embryos confirmed accumulation in parental reproductive tissues and transmission to the next generation. Thus, reproductive dysfunction triggered in parents by PBSA may be vertically transmitted to offspring, leading to persistent developmental toxicity, even in uncontaminated environments. The in silico ECOSAR toxicity prediction performed in this study serves as a conventional single-generation risk reference for PBSA, and this computational outcome is contrasted with our multigenerational in vivo results below. Notably, the in silico ECOSAR prediction indicated low acute toxicity and minimal environmental risk from direct single-generation exposure to PBSA. However, our experimental results demonstrate that significant intergenerational neurodevelopmental toxicity occurs following parental PBSA exposure. This inconsistency highlights the inadequacy of conventional acute toxicity tests and QSAR-based risk assessments in evaluating the comprehensive dangers by environmental contaminants, particularly substances with potent intergenerational toxicity. Hence, it is imperative to incorporate multi- and intergenerational investigations into the environmental risk assessment frameworks for organic UV filters like PBSA.

Notably, parental and F1 continuous exposure to PBSA (F0+/F1+) significantly exacerbated developmental disorders compared to parental exposure alone (F0+/F1−), with more severe developmental defects observed under dual parental and offspring exposure. The adverse phenotypes detected in F1 larvae are attributed to intergenerational effects, influenced by both maternal PBSA chemical translocation and germline epigenetic reprogramming. Maternal PBSA exposure predisposed offspring embryos to a susceptible developmental state through three plausible molecular pathways: altered DNA methylation at Nrf2-target promoters, depletion of antioxidant maternal mRNAs stored in oocytes, and decreased embryonic mitochondrial density. Collectively, these pathways rendered progeny vulnerable to oxidative insults. Meanwhile, this ancestral priming effect follows the DOHaD framework describing prenatal pollutant-induced developmental programming [19]. This ancestral priming effect aligns with findings showing that parental benzotriazole exposure alone led to enduring molecular changes in subsequent unexposed generations of Daphnia magna, suggesting that a single ancestral exposure can heighten descendants’ susceptibility to future stressors [20]. Interestingly, using a distinct two-hit paradigm (first hit in F0, second hit three generations later in F4), Ref. [21] also observed that a second exposure produced behavioral deficits in rats, although direct exposure alone (F1) did not cause observable effects in their model [21]. In contrast to that study, parental PBSA exposure alone (F0+/F1-) already induced measurable developmental disorders, and continuous offspring exposure (F0+/F1+) triggered far more severe developmental damage. Together, these cross-species comparisons demonstrate that while the potency of single-generation effects may vary across toxicants and model systems, the principle that multigenerational exposure amplifies risk appears to be a conserved phenomenon.

F1 larvae directly exposed to PBSA without parental exposure (F0−/F1+) showed minor and mostly insignificant developmental impairments. This comparison confirmed that the significant intergenerational developmental and neurotoxic effects observed in this study were mainly due to parental transmission rather than direct embryonic exposure. Our study demonstrates that PBSA delivered from parents disturbs redox balance, which further leads to defective neurodevelopment in offspring. It is important to note that changes in ROS, MDA, and CAT only exhibit an association with neurodevelopmental defects, rather than establishing a direct causal relationship. Nevertheless, the simultaneous downregulation of neurodevelopmental genes and the reduction in motor neuron axon length, in conjunction with oxidative injury, strongly implies that impaired antioxidant balance is a pivotal factor contributing to intergenerational neurotoxicity in PBSA. Comparable neurodevelopmental abnormalities in progeny have been reported upon parental exposure to environmental pollutants [11,22,23]. These findings extend the toxicological understanding of PBSA, indicating that its adverse effects cover both reproductive endocrine disruption and intergenerational neurodevelopmental impairment.

This study comprehensively investigated the intergenerational neurodevelopmental toxicity of PBSA and its underlying mechanisms. However, certain limitations should be acknowledged. Firstly, this study focused solely on the F1 generation, raising uncertainties about the persistence and heritability of adverse effects in subsequent generations, particularly F2. Distinguishing between intergenerational effects (F1 damage from maternally transferred PBSA) and transgenerational effects (epigenetic abnormalities in F2+ without chemical residue) is crucial. Phenotypic traits in F1 result from both direct maternal PBSA transfer and potential germline epigenetic changes, such as histone modifications and small RNA transmission. Nonetheless, the absence of F2 data hinders definitive confirmation of pure transgenerational inheritance. Secondly, we only measured three oxidative biomarkers (ROS, MDA, and CAT), limiting our ability to establish a definitive causal link between redox imbalance and neural damage. These biomarkers only show correlation rather than causation, future antioxidant rescue tests are needed to confirm if oxidative stress mediates neurodevelopmental toxicity. Further antioxidant rescue assays are necessary to confirm the relationship between oxidative stress and neurodevelopmental defects. Thirdly, while oxidative stress and axonal damage were identified as key pathways, detailed molecular events of parental transmission, such as maternal factor transfer, germline epigenetic modification, and miRNA regulation, remain incompletely understood. Fourthly, neurodevelopmental toxicity in this study was assessed only through motor neuron axon length and a limited set of neurodevelopment-related genes, lacking behavioral functional assessments or additional neural biomarkers. Subsequent investigations will incorporate larval behavioral indicators and more neural biomarkers for enhanced mechanistic insights. Fifthly, insufficient human epidemiological or in vivo data are available on the intergenerational neurotoxicity of PBSA. Zebrafish research implies implications for human prenatal risk evaluation due to widespread PBSA exposure from sunscreens. Zebrafish are a pertinent model for assessing prenatal chemical risks due to shared neural developmental pathways with humans. Concerns arise from observed oxidative and neurodevelopmental damage across generations in zebrafish F1 larvae, suggesting potential interference in fetal neural development in humans due to maternal PBSA exposure. Nevertheless, differences between species in PBSA absorption, metabolism, placental transfer, and antioxidant levels impede direct extrapolation. Further investigations, such as human placental testing, neural differentiation assays, and cohort studies, are crucial for evaluating developmental risks linked to maternal PBSA exposure. Finally, larvae were pooled to secure sufficient tissue for biochemical measurements, precluding individual-level correlation between oxidative stress and neurodevelopmental indicators. Further research involving multigenerational exposure, epigenetic analysis, and rescue experiments is necessary.

## 5. Conclusions

In conclusion, parental life-cycle exposure to environmentally relevant concentrations of PBSA induces significant intergenerational neurodevelopmental toxicity in zebrafish offspring. Continuous PBSA exposure in F1 larvae further exacerbates these toxic outcomes, whereas direct F1 exposure to PBSA results in mild impacts. This study illustrates that oxidative stress imbalance contributes to the neurodevelopmental disorders observed in F1 offspring. Building upon our previously published findings, PBSA-induced parental reproductive endocrine disruption and putatively impaired gamete quality are hypothesized to enhance offspring sensitivity, which may serve as a potential biological basis for the vertical transmission of developmental toxicity. Together, parental reproductive endocrine disruption may act as a predisposing factor, while disrupted redox homeostasis is a prominent accompanying adverse endpoint in PBSA-induced intergenerational neurodevelopmental toxicity in zebrafish larvae. This study offers novel evidence regarding the intergenerational risks associated with UV filters and highlights the importance of incorporating multigenerational effects into environmental risk assessment.

## Figures and Tables

**Table 1:** Primer sequence for RT-qPCR.

Gene Bank Number	Gene	Forward Primer	Reverse Primer	Length
DRU30711	*shha*	GCAAGATAACGCGCAATTCGGAGA	TGCATCTCTGTGTCATGAGCCTGT	117 bp
NM_131341	*gap43*	GCAGCAGGAAGTGGAGAAGCCA	GGATTCCTCAGCAGCGTCTGGT	181 bp
NM_194388	*α-tublin*	GGATTTGCCCAACAGGA	CGCACCTCATCAATGACAGTGG	204 bp
AY860977	*mbp*	AATCAGCAGGTTCTTCGGAGGAGA	AAGAAATGCACGACAGGGTTGACG	138 bp
NM_181601	*β-αctin*	AAGCAGGAGTACGATGAGTC	TGGAGTCCTCAGATGCATTG	238 bp

**Table 2:** In silico toxicity prediction of PBSA using ECOSAR and Toxtree.

Parameters	Specifications
Chemical name	2-Phenylbenzimidazole-5-sulfonic acid
Molecular weight	274.3
Estimated log Kow	−0.1594
Estimated water solubility (mg/L) ^a^	23,630
LC_50_ for 96 h for fish (mg/L) ^a^	1.96 × 10^5^
LC_50_ for 48 h for daphnid (mg/L) ^a^	9.12 × 10^4^
EC_50_ for 96 h for green algae (mg/L) ^a^	2.98 × 10^4^
Verhaar scheme for predicting toxicity-mode of action ^b^	Class 5 (not possible to classify according to these rules)
Kroes TTC decision tree ^b^	Substance would not be expected to be a safety concern
Cramer rules ^b^	Class I (low)
Michael acceptor ^b^	Not reactive via Michael addition
Structure alerts for the in vivo micronucleus assay in rodents ^b^	At least one positive structural alert for the micronucleus assay (Class I)
Skin sensitization alerts	Alert for Acyl Transfer agent identified
DNA binding ^b^	Alert for Michael acceptor identified
Protein binding ^b^	Alert for Michael acceptor identified
Benigni/Bossa rules for carcinogenicity and mutagenicity ^b^	Structural alert for non-genotoxic carcinogenicity Negative for genotoxic carcinogenicity
In vitro mutagenicity (Ames test) alerts by ISS ^b^	No alerts for S. typhimurium mutagenicity

^a^ ECOlogical Structure–Activity Relationship (ECOSAR) model version 2.2 baseline toxicity was used to determine toxicity prediction. ^b^ Toxtree-Toxic Hazard Estimation by decision tree approach. Different prediction modules in Toxtree employ independent structural alert rules, which may lead to divergent outputs for Michael acceptor-related endpoints.

## Data Availability

The original contributions presented in this study are included in the article/Supplementary Materials. Further inquiries can be directed to the corresponding authors.

## Supplementary Materials

The following supporting information can be downloaded at: https://www.mdpi.com/article/10.3390/toxics14070639/s1, Table S1: Nominal and measured concentrations of PBSA in zebrafish; Supplementary Materials Text S1: LC-MS detection method for PBSA analysis.
