# Supplementary material for: Parental Lifetime PBSA Exposure Induces Neurodevelopmental Toxicity in F1 Zebrafish
Source: Toxics. 2026 Jul 22;14(7):639. doi: 10.3390/toxics14070639 (PMC13417377; doi:10.3390/toxics14070639)
Supplement: Supplementary file 1 [file toxics-14-00639-s001.zip › toxics-4371288-supplementary.pdf]

Supplementary Materials

Text S1. *Liquid chromatography–mass spectrometry (LC-MS)*

Samples were homogenized in 100 μL of LC-MS grade methanol, mixed with ultrapure water to a 7:3 ratio (methanol:water), ultrasonicated for 15 min, incubated at 4 °C for 1 hour, and centrifuged. The supernatant was dried under nitrogen and resuspended in 7:3 methanol/water for analysis. LC-MS analysis was performed on an AB 4500 QTRAP system with a Titank-F5 column (2.1 × 100 mm, 1.8 μm). The mobile phase consisted of water containing 20 mM of ammonium acetate and 0.1% of formic acid (A) and methanol (B) at 0.3 mL/min. The gradient program was: 100% A for 0.5 min, to 70% A over 0.5 min, to 20% A over 2 min (held for 2 min), and then back to 100% A within 0.1 min and held for 0.9 min (total 6 min). The column temperature was 40 °C, and positive ion mode was used (Q1/Q3: 275/194 and 166). The method showed good linearity (0-32 μg/L, R^2=0.9985), recovery rates of 99.8%–113.9%, intra- and inter-day precision of 0.7-3.6% and 1.5-3.9%, respectively, and LOD/LOQ of 0.5/1.65 μg/L.

Table S1. Nominal and measured concentrations of PBSA in zebrafish.

Organization	F0/F1	Nominal concentration (μg/L)	Measured concentration (μg/mg)
Ovaries	F0	0	ND
Ovaries	F0	0.2	0.05 ± 0.002
Ovaries	F0	2	0.06 ± 0.001
Ovaries	F0	20	1.81 ± 0.030
Testis	F0	0	ND
Testis	F0	0.2	0.14 ± 0.020
Testis	F0	2	0.17 ± 0.012
Testis	F0	20	1.24 ± 0.080
Embryo (2 hpf)	F1	0	ND
Embryo (2 hpf)	F1	0.2	0.004 ± 0.0001
Embryo (2 hpf)	F1	2	0.008 ± 0.0002
Embryo (2 hpf)	F1	20	1.01 ± 0.05

The data are expressed as the mean ± SD of three replicates. ND: Not detected.
